# H3K18la-PSMG1 Axis in Bladder Cancer Progression: Curcumin as a Therapeutic Candidate

**DOI:** 10.7150/ijbs.135180

**Published:** 2026-05-29

**Authors:** Zhe Yu, Jinge Zhang, Zihuan Wang, Shu Wei, Chen Chen, Yuan Huang, Qin Fan, Fan Deng, Haiyong Chen, Zhangfeng Zhong, Lina Hou, Wanlong Tan, Fei Li

**Affiliations:** 1 Department of Urology, Nanfang Hospital, Southern Medical University, Guangzhou, Guangdong 510515, P. R. China.; 2 Department of Urology, Baoshan People's Hospital, Baoshan, Yunnan 678000, P. R. China.; 3 School of Traditional Chinese Medicine, Southern Medical University, Guangzhou, Guangdong 510515, P. R. China.; 4 Department of Cell Biology, School of Basic Medical Sciences, Southern Medical University, Guangzhou, Guangdong 510515, P. R. China.; 5 School of Chinese Medicine, LKS Faculty of Medicine, The University of Hong Kong R619, 3 Sassoon Road, Pokfulam, Hong Kong, SAR, 999077, P. R. China.; 6 Macao Centre for Research and Development in Chinese Medicine, Institute of Chinese Medical Sciences, University of Macau, Macao, SAR, 999078, P. R. China.; 7 Huiqiao Medical Center, Nanfang Hospital, Southern Medical University, Guangzhou, Guangdong 510515, P. R. China.

**Keywords:** bladder cancer, curcumin, H3K18la, PSMG1, single cell

## Abstract

Although multiple therapeutic modalities, including surgery, chemotherapy, radiotherapy, immunotherapy, and targeted therapy, have improved the management of bladder cancer, the clinical outcome of muscle-invasive bladder cancer (MIBC) remains unsatisfactory. To address this challenge, we identified MIBC-related genes (MIBC.RGs) through transcriptomic and proteomic analyses and developed a prognostic model to predict patient outcomes. Among the candidate genes, PSMG1 was prioritized through an integrated framework combining machine learning-based screening and single-cell transcriptomic analysis. Experimental analyses revealed that PSMG1 was markedly upregulated in bladder cancer (BCa), progressively upregulated from normal tissue to MIBC, and PSMG1 silencing reduced cell proliferation, invasion, and clonogenic capacity in vitro, while attenuating tumor growth in vivo. Mechanistically, our data suggest that PSMG1 may promote BCa aggressiveness, at least in part, by affecting E-cadherin stability and EMT-related signaling. Epigenetic profiling revealed significant H3K18la enrichment at the PSMG1 promoter, supporting a potential H3K18la-PSMG1 regulatory axis. Finally, molecular docking, proteomic profiling, and Drug Affinity Responsive Target Stability (DARTS) assays prioritized Curcumin as a candidate compound potentially associated with PSMG1 targeting. Overall, our findings indicate that the H3K18la-PSMG1 axis may participate in BCa progression and support further evaluation of Curcumin in PSMG1-associated therapeutic strategies.

## Introduction

Bladder cancer (BCa) represents a major cause of cancer-associated mortality and ranked fourth in incidence among men in 2023^1^, ^2^. According to tumor invasion depth, BCa is broadly classified as either non-muscle-invasive (NMIBC) or muscle-invasive (MIBC)—two subtypes that differ markedly in clinical course and progression risk^3^, ^4^. Although treatment approaches for MIBC have expanded, including surgery, systemic therapy, radiotherapy, immunotherapy, and targeted therapy, effective disease control remains difficult. For patients with MIBC undergoing radical cystectomy, the 5-year survival rate is approximately 50-60%; however, it declines to 10-15% in the presence of lymph node involvement or metastatic disease^5^-^7^.

Tumor suppressor gene alterations (e.g., TP53, RB1, PTEN) commonly occur in MIBC and drive malignant progression by disrupting cell-cycle control and signaling networks^8^. In addition, the frequent development of drug resistance, particularly to cisplatin-based chemotherapy, further limits treatment efficacy. Resistance mechanisms include enhanced DNA damage repair, evasion of apoptosis, and metabolic reprogramming^9^, ^10^. MIBC is also defined by strong invasive and metastatic potential, closely tied to epithelial-mesenchymal transition (EMT) activation and the tumor immune microenvironment's remodeling. These unresolved clinical problems emphasize the need to discover additional molecular drivers and actionable therapeutic targets for MIBC.

Advances in multi-omics and gene editing technologies have revolutionized cancer research^11^, ^12^. Bulk RNA sequencing (RNA-seq) is widely used to characterize overall transcriptomic landscapes^13^, while single-cell RNA sequencing (scRNA-seq) resolves transcriptional differences across individual cell populations^14^. Proteomics provides insight into protein function and regulation^15^, and CRISPR-Cas9 technology now provides a powerful platform for interrogating gene function in functional genomics studies^16^. The integration of multi-omics data provides a critical link between molecular mechanisms and clinical translation, offering a robust foundation for precision oncology.

Machine learning and artificial intelligence have significantly enhanced biomarker discovery, prognostic modeling, and therapeutic target identification in cancer research^17^, ^18^. Machine learning offers advantages over conventional statistical approaches by enabling the detection of complex nonlinear associations within high-dimensional transcriptomic and proteomic data, which can improve feature selection and the development of predictive models. By integrating multiple algorithms^19^, we improved gene screening and prognostic model development for BCa.

Here, we used an integrated multi-omics strategy to define muscle-invasive bladder cancer-related genes (MIBC.RGs) and identified PSMG1 as a critical factor associated with disease progression from normal bladder tissue through NMIBC to MIBC. We further investigated a potential relationship between H3K18la and PSMG1 expression in association with EMT-related phenotypes during BCa progression. In addition, we explored candidate compounds associated with PSMG1-related therapeutic targeting, including Curcumin. Together, these analyses provide a basis for further investigation of the H3K18la-PSMG1 axis and related therapeutic strategies in BCa.

## Methods

### Data collection and acquisition

To define MIBC-related genes (MIBC.RGs), bulk RNA-seq data were collected from two GEO datasets, GSE32548 and GSE32894. The proteomic profiles came from the study conducted by Ning Xu *et al*.^20^. The TCGA-BLCA dataset from The Cancer Genome Atlas (TCGA) served as the training cohort, whereas five GEO datasets, GSE13507, GSE32548, GSE32894, GSE39281, and GSE48075, were used for external validation. Single-cell transcriptomic analyses focusing on PSMG1 were conducted using the GSE129845, GSE130001, GSM4006644, GSE192575, and GSE211388 datasets. Additional data for PSMG1-related analyses were collected from publicly available resources, including BEST^21^, Sangerbox^22^, SolvingLab, and DepMap^23^. Sangerbox^22^ was used for downstream evaluations, including AUC estimation, Kaplan-Meier survival analysis, clinicopathological association analysis, immune infiltration characterization, and mutation profiling. Immune-cell abundance was further estimated with CIBERSORT, followed by Wilcoxon rank-sum testing to assess differences between the high- and low-risk groups.

Normalization of bulk RNA-seq profiles was conducted with the DESeq2 package^24^, with size factor estimation applied to adjust for differences in sequencing depth. ScRNA-seq data underwent quality control using Seurat^25^, low-quality cells were removed if they contained fewer than 200 detected genes or exhibited mitochondrial gene proportions above 10%. Batch effects across multiple datasets were corrected using the Harmony^26^ package.

### MIBC.RGs were selected through transcriptomic and proteomic analyses

We separately applied WGCNA^27^ to the GSE32548 and GSE32894 datasets to identify MIBC-associated gene modules. The genes within these two modules were combined to form Geneset1 (Table S1). Proteomic profiles were analyzed with the R package DESeq2 to identify genes significantly upregulated in MIBC relative to NMIBC samples, applying a cutoff of log2FC > 1.2 and *P* < 0.05. The resulting gene set was defined as Geneset2 (Table S2). The intersection of Geneset1 and Geneset2 generated the final set of MIBC.RGs (Table S3).

### Integration of multiple machine learning algorithms for prognostic model construction

The MIBC.RGs underwent univariate Cox regression analysis to generate the candidate gene set for model construction (Table S4). Model construction and feature selection were subsequently carried out using an integrated machine-learning framework comprising 101 algorithmic combinations generated from ten survival modeling strategies, including RSF and GBM as tree/boosting-based methods; Enet, LASSO, ridge, and stepwise Cox as Cox regression-based approaches; CoxBoost as a boosting-based Cox model; plsRcox and SPCA as dimension-reduction-based survival models; and survival-SVM as a kernel-based survival learning method^19^. To improve model robustness, each algorithm generated an independent feature ranking, and genes consistently retained across multiple algorithms were prioritized for downstream modeling. Concordance index (C-index) performance across the training and validation cohorts primarily guided the selection of the final model. Among all 101 algorithmic combinations, Ridge regression achieved the highest C-index (0.673) and showed stable performance in external validation datasets; therefore, we selected it as the final prognostic model (Figure 2A). Given the high dimensionality and potential collinearity of transcriptomic features, the regularization property of Ridge regression further supported its suitability for prognostic modeling in this setting. The final Ridge-based model included 34 genes (Table S5). A detailed description of the model selection strategy, algorithm integration framework, and validation procedure is provided in Supplementary Material 1.

Ten-fold cross-validation, which randomly split the dataset into training (90%) and testing (10%) subsets in each iteration, was used to assess the robustness of our prognostic model. In addition, external validation was conducted using five independent GEO datasets (GSE13507, GSE32548, GSE32894, GSE39281, and GSE48075). Model performance was assessed with the C-index, Kaplan-Meier survival analysis and time-dependent ROC curves (AUC). Calibration plots examined the concordance between predicted and observed survival probabilities.

### PSMG1 was selected as a key driver in BCa progression based on pseudo-time analysis

The R packages Seurat, SingleR^28^, and Harmony performed dimensionality reduction, clustering, annotation, and subdivision of epithelial subpopulations in scRNA-seq data. For pseudo-time analysis, we employed the R package Monocle3^29^. Initially, uniCox analysis was applied to model genes (Table S6), resulting in the selection of 32 genes with hazard ratios (HR) greater than 1.0. Their temporal trajectories were visualized in the scRNA-seq data. Subsequently, 10 genes demonstrating robust trends from normal tissue to MIBC were chosen. Finally, PSMG1, which exhibited the highest HR of 1.35, was selected for further analysis.

Spatial transcriptomic data were obtained from the study by Kenneth H. Gouin III *et al*.^30^, which were analyzed using STOmicsDB^31^. Gene Set Cancer Analysis (GSCA)^32^, ^33^ was applied to assess whether PSMG1 expression varied according to bladder cancer stage.

### A comprehensive analysis of PSMG1 was performed to explore its role in BCa progression

Pan-cancer analysis of PSMG1 was conducted using Sangerbox. Survival analysis was performed through SolvingLab. Correlation analyses with various clinical features were completed using BEST. Representative immunohistochemical staining data for PSMG1 were accessed through The Human Protein Atlas (HPA)^34^. Multi-omics enrichment analyses were conducted employing BEST and ClusterProfiler^35^. Predictive analysis of PSMG1's impact on BCa cell functions was performed using DepMap.

### The role of PSMG1 in promoting BCa progression was validated

Lentiviral shRNA constructs were obtained, and UMUC3 cells were transduced with lentiviral particles to generate stable cell lines as per the manufacturer's protocols. Quantitative PCR (qPCR), CCK-8, colony formation, and subcutaneous tumorigenesis experiments were performed as reported in a prior study^36^. The Transwell assay was conducted following the methodology outlined by Li Yi *et al*.^37^. Changes in the protein expression levels of EMT marker genes, including N-cadherin, Vimentin, SNAIL1, and E-cadherin, following PSMG1 knockdown were validated by Western blotting. The sequences of the shPSMG1 constructs were GTCGACATGTTACCGATTATA and GCAATTCTGTACTTGTGTTAT.

A cycloheximide (CHX) chase assay was used to evaluate E-cadherin protein stability. Following exposure to cycloheximide (50 µg mL⁻¹), UMUC3 cells were harvested at 0, 3, 6, 9, and 12 h. E-cadherin levels were assessed via Western blotting with Actin as the loading control, and representative blots are displayed.

### The regulation of PSMG1 by H3K18la was validated

The potential interaction between H3K18la and PSMG1 was investigated using ATAC-seq and ChIP-seq^36^. Additionally, potential interacting proteins of PSMG1 were predicted using the AlphaFold3 artificial intelligence algorithm^38^. Changes in PSMG1 expression following treatment with histone lactylation inhibitors 2-DG and c646, as well as the histone lactylation promoter Nala, were validated through qPCR and Western blotting. Detailed experimental procedures are provided in a previous study^36^.

### Potential drugs targeting PSMG1 were explored

Candidate drugs targeting PSMG1 were identified using the Beyondcell algorithm^39^, with inclusion criteria defined as drug sensitivity showing a significant positive correlation with PSMG1 expression levels (cor > 0.2, *P* < 0.001). Six compounds meeting these criteria—Curcumin, Emodin, Apigenin, Naringenin, Taxifolin, and Vitexin—were shortlisted based on favorable molecular docking scores obtained using SwissDock^40^, which evaluated their binding poses and interaction energies with PSMG1. To further evaluate potential compound-protein interactions, Drug Affinity Responsive Target Stability (DARTS) assays were conducted with proteinase K digestion, followed by Coomassie blue staining and Western blotting. Comparative analyses incorporated both docking results and pharmacological data. Although Emodin exhibited the lowest binding energy, Curcumin demonstrated superior binding stability in DARTS assays, along with extensive evidence of anticancer efficacy and a well-established safety profile. Other candidates, while promising in silico, lacked comparable validation in terms of stability or safety and were therefore not advanced to further experimental evaluation. Therefore, Curcumin was prioritized as the primary candidate for subsequent experimental validation. Sequencing data from Curcumin-treated cell lines are provided in Supplementary Material 2.

### Data statistics and analysis

Statistical analyses were conducted with R version 4.2.1 and GraphPad Prism 10, and all in vitro experiments (unless otherwise noted) were independently repeated three or more times. Quantitative data are presented as mean ± SD. Comparisons between two groups were performed using a two-tailed Student's t test, whereas comparisons among multiple groups were performed using one-way ANOVA followed by Tukey's multiple-comparison test. Kaplan-Meier survival curves were compared using the log-rank test. A *P* value < 0.05 was considered statistically significant. For Western blot-based assays, including CHX chase and DARTS, representative results are shown.

## Results

### The acquisition of MIBC.RGs was performed through integration of multiple data sources

**Figure 1:**
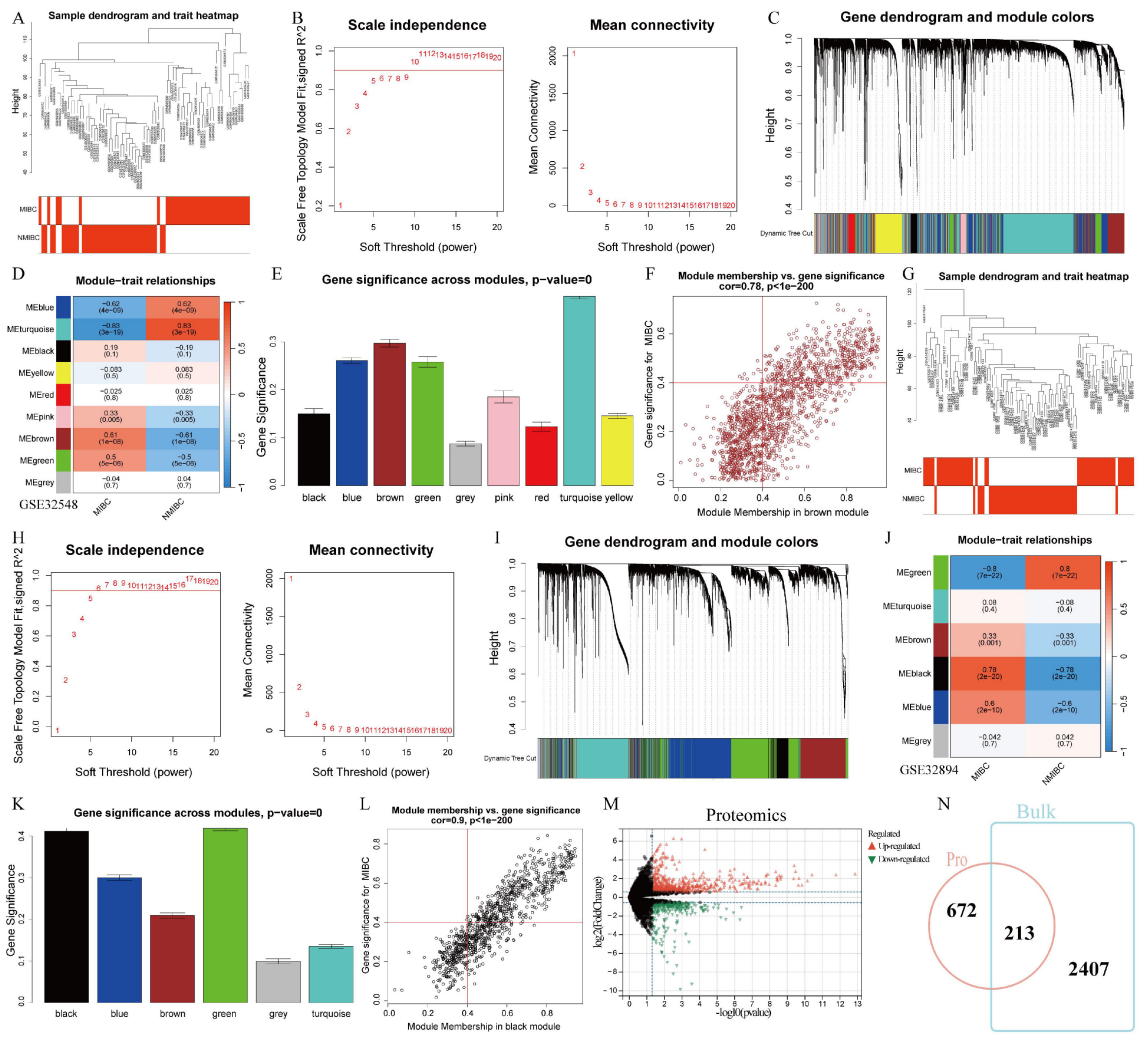
The acquisition of MIBC.RGs. **(A)** The sample dendrogram and trait heatmap of the GSE32548 dataset. **(B)** Analysis of the scale-free index for various soft-threshold powers. **(C)** The dynamic tree depicting the gene dendrogram and module colors. **(D)** Module-trait relationships. **(E)** Gene significance across modules. **(F)** The relationship between brown module membership and gene significance. **(G)** The sample dendrogram and trait heatmap of the GSE32894 dataset. **(H)** Scale-free topology fit index across different soft-thresholding powers.** (I)** Gene dendrogram and module colors shown in the dynamic tree. **(J)** Module-trait relationships. **(K)** Gene significance across modules. **(L)** Correlation of black module membership with gene significance. **(M)** Differential gene analysis between MIBC and NMIBC based on proteomic data. **(N)** Intersection analysis of MIBC-related genes between transcriptomics and proteomics datasets.

To comprehensively identify factors driving MIBC in the central pathway, bulk RNA-seq and proteomics data were integrated. Initially, two bulk RNA-seq datasets with comprehensive clinical data were included. In dataset GSE32548, WGCNA identified the module most strongly connected with MIBC. After plotting the distribution of MIBC and NMIBC samples (Figure 1A), scale independence analysis determined a Soft Threshold power of 9 (Figure 1B). Modules were color-coded (Figure 1C). The brown module showed the strongest correlation with MIBC (Figure 1D). The genes corresponding to this module were then extracted. This module also displayed greater gene significance in both overall and MIBC samples (Figure 1E, F), confirming the validity of the analysis. The same methodology was applied to GSE32894 (Figure 1G-L). The union of MIBC-related genes from both bulk RNA-seq datasets formed Geneset1 (Table S1).

Next, differential genes between MIBC and NMIBC samples were identified from proteomics data, retaining only upregulated genes in MIBC, resulting in Geneset2 (Table S2) (Figure 1M). The intersection of Geneset1 and Geneset2 from both omics analyses yielded the final set of MIBC.RGs (Table S3) (Figure 1N).

### A prognostic model was constructed and gene screening was performed using multiple machine learning integration algorithms

**Figure 2:**
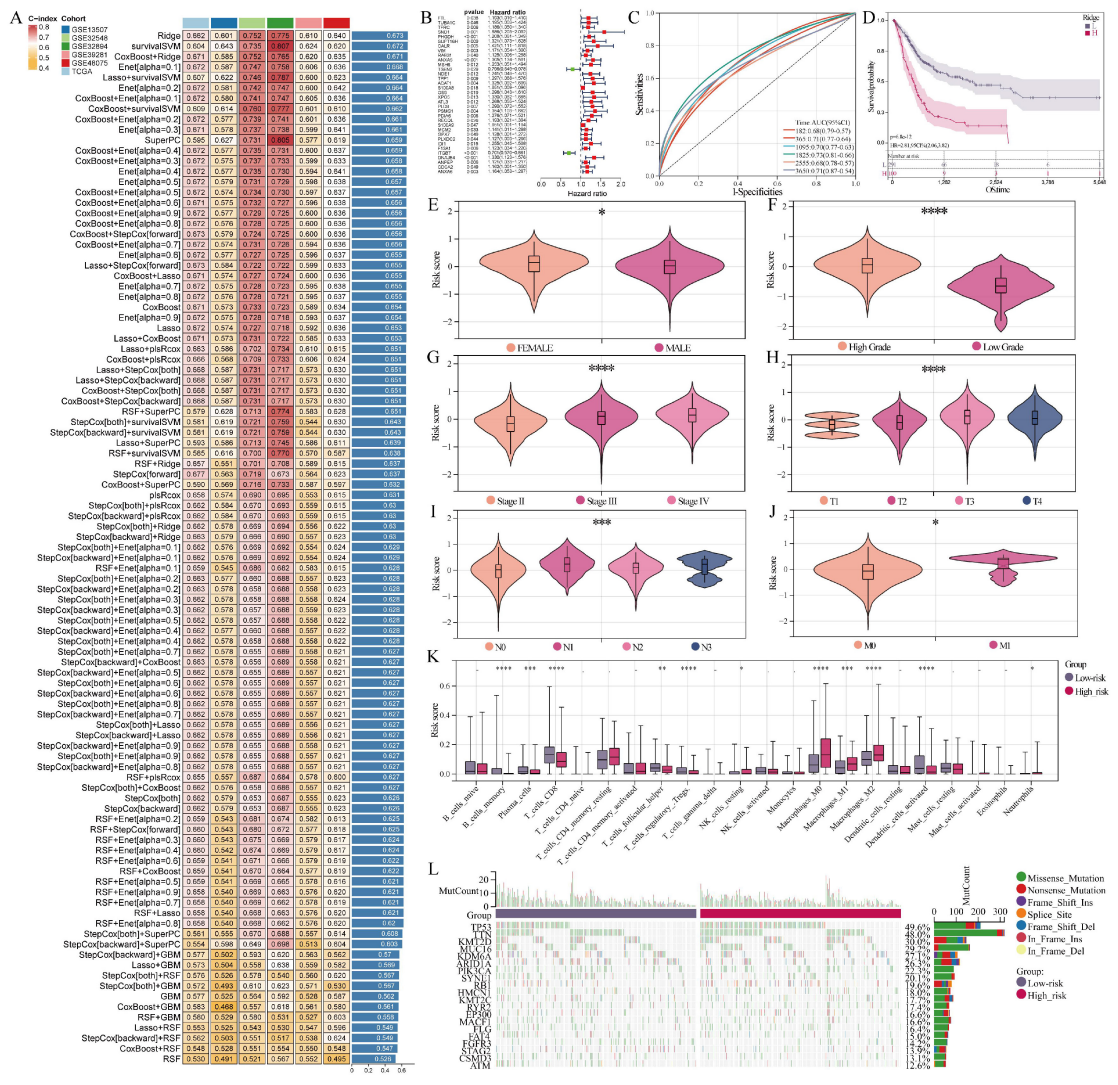
Multiple machine learning-integrated analysis. **(A)** Heatmap of the C-index for 101 algorithm combinations. The Ridge-based model, having the highest C-index, was chosen as the final model. **(B)** UniCox regression analysis of model genes. **(C)** ROC curve based on the model. **(D)** Survival analysis between the high- and low-risk groups. **(E-J)** Violin plots showing the correlation of risk score with gender, grade, stage, T stage, N stage, and M stage. **(K)** Box plots were used to compare immune-cell infiltration levels between the high- and low-risk groups, with infiltration scores estimated by CIBERSORT. Statistical significance was assessed using the Wilcoxon rank-sum test. *P < 0.05, **P < 0.01, ***P < 0.001, ****P < 0.0001. **(L)** Waterfall plot illustrating the somatic mutation profiles of the high- and low-risk groups.

Advancements in machine learning algorithms have greatly enhanced the ability to screen gene sets and construct prognostic models based on specific gene expression profiles^19^. From MIBC.RGs, a prognostic model was built by combining multiple machine learning algorithms (Figure 2A, B). We selected the Ridge algorithm from 101 combinations of 10 algorithms, as it achieved the highest concordance index (C-index) of 0.673. The model effectively predicted survival outcomes for BCa patients across six intervals (Figure 2C). We stratified patients into high- and low-risk groups using risk scores, where higher scores correlated with worse prognosis (Figure 2D).

Further analyses incorporated various clinical characteristics. In the female population, a higher risk score was observed (Figure 2E). Notably, elevated risk scores were associated with advanced BCa progression and metastasis (Figure 2F-J). In addition, comparative immune infiltration profiling showed that the high- and low-risk groups were associated with distinct immune-cell composition patterns (Figure 2K). Specifically, the low-risk group exhibited increased CD8+ T-cell infiltration, while the high-risk group showed greater M2 macrophage enrichment. CD8+ T cells are crucial for host defense and tumor cell cytotoxicity^41^, while M2 macrophages are associated with pro-tumorigenic effects^42^. These findings suggest that the MIBC.RG-based risk pattern may be associated with differences in the tumor immune microenvironment. Furthermore, the high-risk group exhibited a more active mutation landscape, including a higher frequency of TP53 mutations (Figure 2L).

In conclusion, a prognostic model based on MIBC.RGs was developed by integrating multiple machine learning algorithms. The model effectively predicted BCa patient outcomes and provided insights into patient progression and metastasis through risk score stratification.

### PSMG1 was selected as a key driver in BCa progression

ScRNA-seq is a powerful tool for uncovering the heterogeneity of transcriptional profiles at the single-cell level, enabling the identification of distinct cell types and functions within specific tissues^43^. One analysis method used in this technique is pseudo-time analysis, which reconstructs the trajectory of cellular changes over time. This approach maps the dynamic progression of cellular states by considering the relative timing of gene expression changes between individual cells, allowing researchers to track how cells transition from one state to another^44^. Leveraging this approach, comprehensive scRNA-seq data for BCa were collected to investigate mechanisms driving BCa progression.

**Figure 3:**
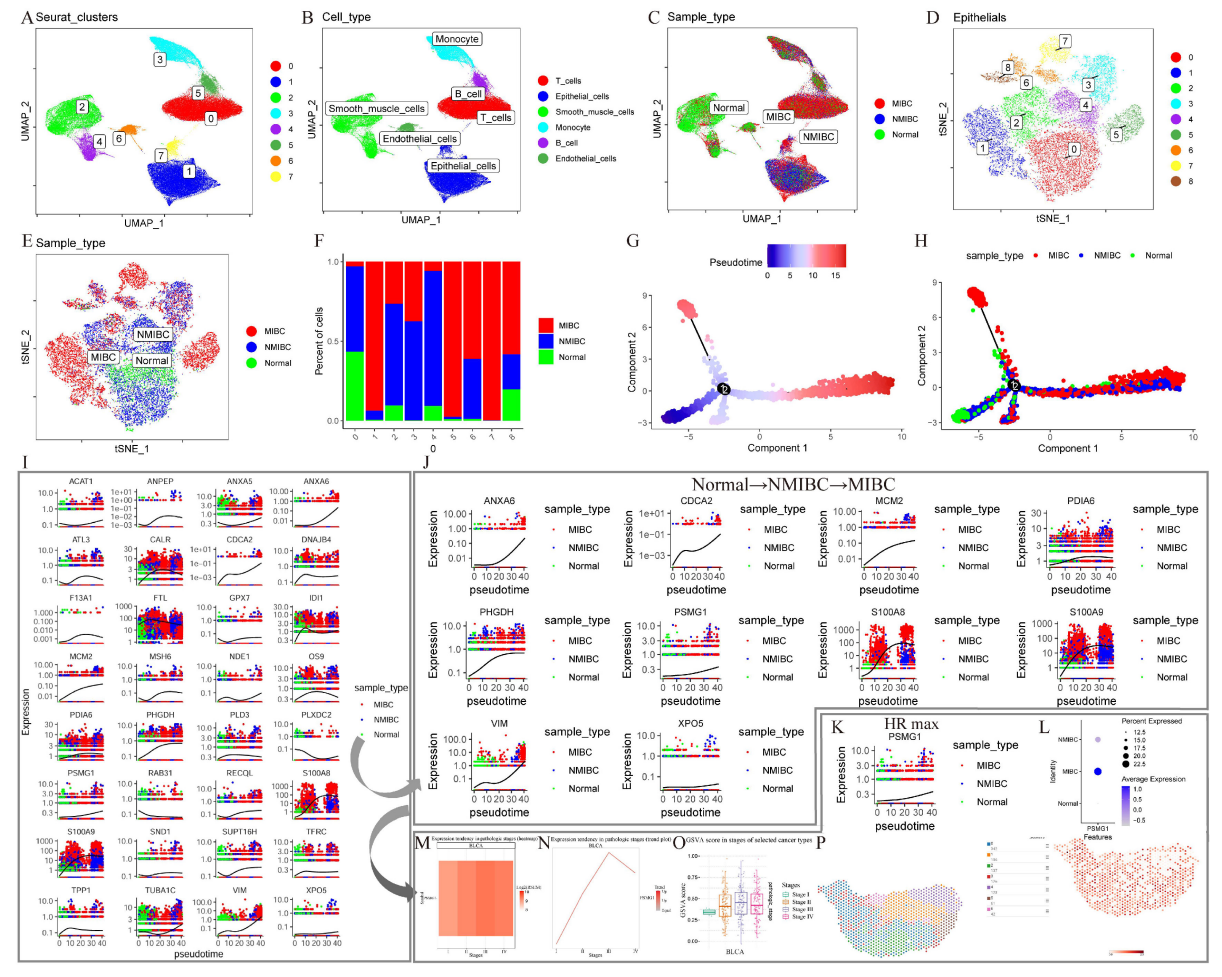
Identification of PSMG1. **(A)** UMAP plot of single-cell clustering of BCa samples. **(B)** UMAP plot annotated with cell clusters. **(C)** Projection of three sample types across various cell clusters. **(D)** Subdivision of epithelial cell cluster into subgroups. **(E)** Projection of three sample types across various epithelial subgroups. **(F)** Proportions of three sample types across various epithelial subgroups. **(G)** UMAP plot showing pseudo-temporal trajectory changes of epithelial subgroups. **(H)** Pseudo-temporal trajectory UMAP plot incorporating sample type information. **(I)** Pseudo-temporal trajectory plots of 32 model genes with HR > 1.0 in uniCox analysis. **(J)** Ten model genes exhibiting a trend from normal to MIBC in expression. **(K)** Pseudo-temporal trajectory plot of PSMG1. **(L)** Bubble plot of PSMG1 expression across different sample types. **(M)** Heatmap of PSMG1 expression levels across different stages. **(N)** Trend plot of PSMG1 expression levels across different stages. **(O)** Box plot of PSMG1 scores across different stages. **(P)** Left: spatial transcriptomic clustering of MIBC data. Right: projection of PSMG1 expression in MIBC spatial transcriptomic data.

After data processing steps including dimensionality reduction, clustering, and annotation, we generated a single-cell atlas of BCa (Figure 3A-C). Special focus was placed on epithelial clusters, which were further subdivided into distinct subpopulations (Figure 3D). Among these, epithelial clusters 1, 5, and 7 exhibited a higher proportion of MIBC cells (Figure 3E, F). Pseudo-time analysis reconstructed cellular trajectories, illustrating transitions from normal tissue to NMIBC and eventually to MIBC (Figure 3G, H). Model genes identified as risk factors were mapped onto pseudo-time trajectories (Figure 3I), and those displaying robust trends from normal tissue to MIBC were further analyzed (Figure 3J).

From this analysis, PSMG1, which had the highest HR of 1.35, was identified as a key focus for subsequent investigations. Elevated expression of PSMG1 in MIBC was validated using scRNA-seq, bulk RNA-seq, and spatial transcriptomic analyses (Figure 3K-P).

In conclusion, through an integrative multi-omics analysis of MIBC.RGs, a prognostic model was constructed and gene selection was performed using advanced machine learning algorithms. By mapping cellular trajectories within the BCa single-cell atlas, PSMG1 was identified as a potential key regulator influencing BCa progression.

### BCa progression was promoted by PSMG1

**Figure 4:**
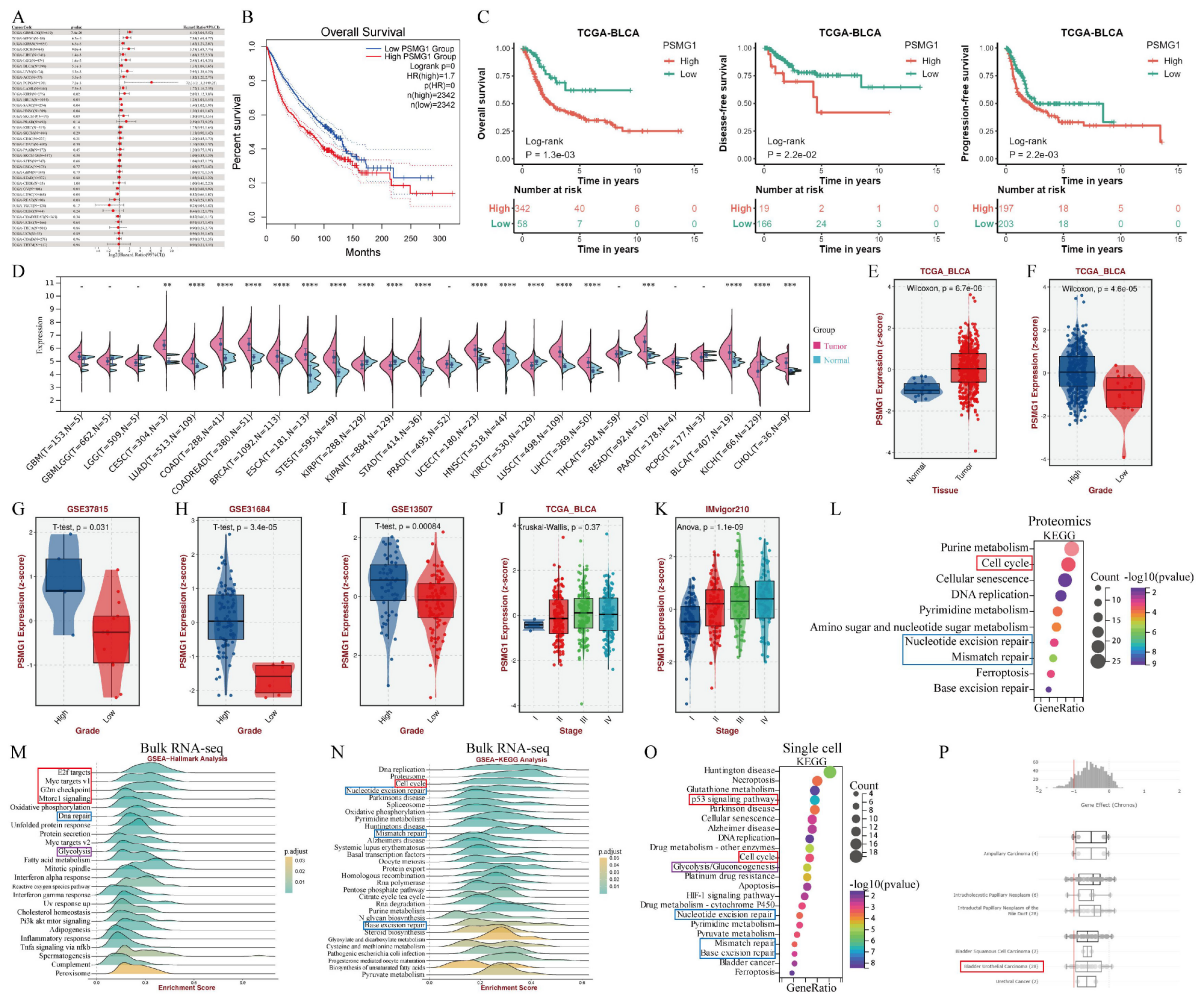
The comprehensive analysis of PSMG1. **(A)** Pan-cancer uniCox regression analysis of PSMG1. **(B)** Large-scale survival analysis integrating tumor cohorts where PSMG1 is considered an independent risk factor. **(C)** Analysis of PSMG1 in BCa for OS (left one), DFS (left two), and PFS (left three). **(D)** Violin plot of pan-cancer expression levels of PSMG1. **(E)** Box plot of PSMG1 expression levels in BCa. **(F-I)** Box plots showing the correlation between PSMG1 expression levels and grade. **(J-K)** Box plots illustrating the correlation between PSMG1 expression levels and stage. **(L)** KEGG pathway enrichment analysis of proteins positively correlated with PSMG1 in the proteomic dataset. **(M)** Hallmark pathway enrichment analysis of PSMG1-associated genes in the bulk RNA-seq dataset. **(N)** KEGG pathway enrichment analysis of PSMG1-correlated genes identified via bulk RNA-seq. **(O)** KEGG pathway enrichment analysis of genes positively correlated with PSMG1 at the single-cell RNA level. Colored boxes indicate recurrently enriched pathways shared across multiple datasets, highlighting the cross-platform consistency of PSMG1-related biological programs. **(P)** Prediction of PSMG1 gene effects based on DepMap.

Currently, research on PSMG1 is limited, with only a few reports in inflammatory bowel disease (IBD), and studies in tumors are scarce. To explore the role of PSMG1 in cancer, particularly in BCa, we conducted further comprehensive analysis. Intriguingly, PSMG1 was identified as an independent risk factor across various cancers (Figure 4A). Large-scale pan-cancer survival analysis demonstrated that high PSMG1 expression was significantly associated with poor prognosis (Figure 4B). High PSMG1 expression was associated with worse overall survival (OS), disease-free survival (DFS), and progression-free survival (PFS) in BCa patients compared to those with low expression (Figure 4C). Beyond BCa, PSMG1 was found to be upregulated in a range of cancer types, reinforcing its association with malignancy (Figure 4D).

BCa tumor tissues showed significantly higher PSMG1 expression than normal tissues (Figure 4E; Figure S1). Moreover, elevated PSMG1 expression correlated with higher malignancy grades in BCa (Figures 4F-K). Functional enrichment analyses of PSMG1 were performed across proteomic, bulk RNA-seq, and single-cell RNA-seq datasets to evaluate the robustness and consistency of its associated biological programs from multiple molecular layers. As shown in Figure 4L-O, these independent analyses convergently highlighted pathways related to tumor proliferation and progression, including cell cycle, E2F/MYC-related programs, G2/M checkpoint, mTORC1 signaling, and p53 signaling^45^-^50^. In addition, pathways associated with DNA damage repair and glycolytic metabolism were also recurrently enriched across datasets. Collectively, these findings support the cross-platform consistency of PSMG1-associated biological programs. Analysis using DepMap predicted a gene effect score < 0, indicating that PSMG1 knockdown or inhibition could suppress BCa cell proliferation (Figure 4P).

In summary, these findings underscored the critical role of PSMG1 in promoting BCa progression and highlighted its potential as a therapeutic target.

### The role of PSMG1 in promoting BCa progression was validated

**Figure 5:**
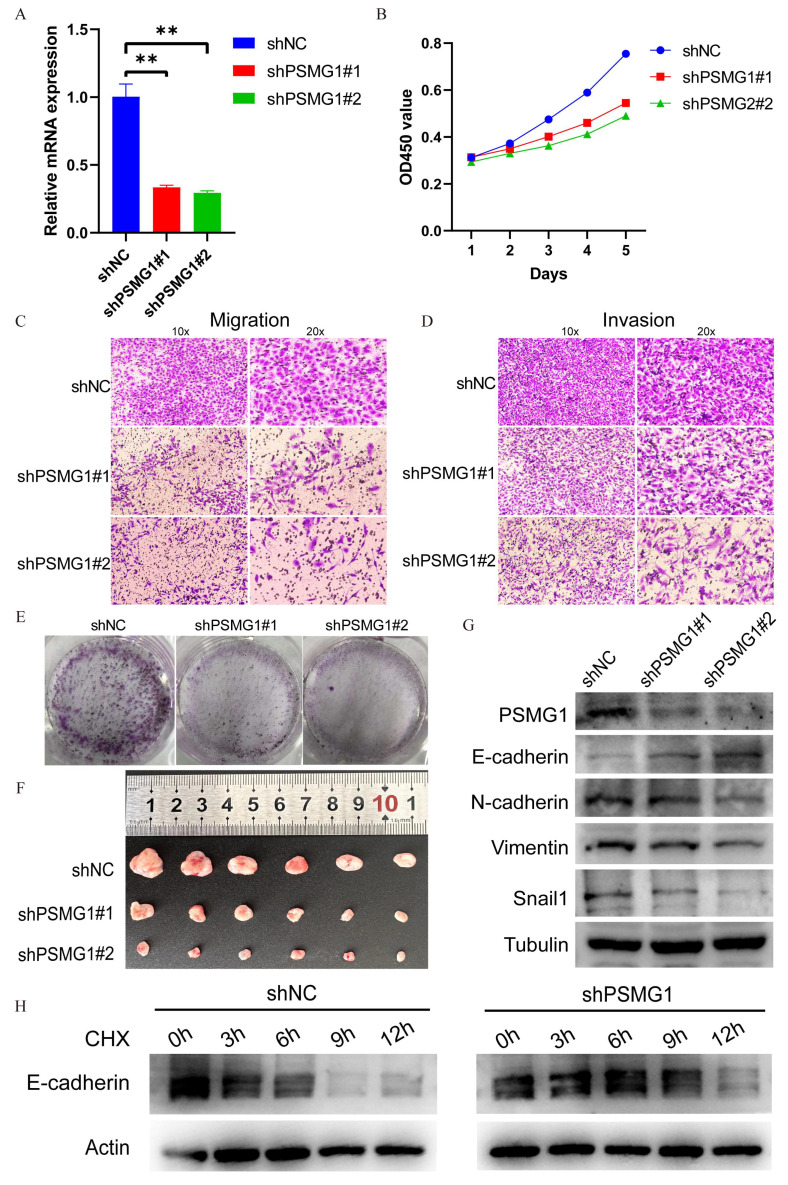
Validation of BCa progression promoted by PSMG1. **(A)** PSMG1 was knocked down using shRNA technology, and the effectiveness of knockdown was validated by qPCR. *: *P* < 0.05, **: *P* < 0.01, ***: *P* < 0.001. **(B)** Assessment of proliferative function by CCK-8 assay. **(C-D)** Evaluation of migratory and invasive abilities using Transwell assays. **(E)** Colony formation assay reflecting proliferative function changes. **(F)** Subcutaneous tumorigenesis assay in mice. **(G)** Western blotting assessed EMT marker expression (N-cadherin, Vimentin, SNAIL, E-cadherin) after PSMG1 knockdown. **(H)** CHX-chase Western blotting was performed in the control group (shNC) and the PSMG1 knockdown group (shPSMG1). Cells were treated with 50 µg mL^-^¹ CHX and harvested at 0, 3, 6, 9, and 12 h. E-cadherin, PSMG1, and Actin were detected, with Actin used as the loading control. Representative results are shown.

To validate PSMG1's role in promoting BCa progression, we knocked down PSMG1 using shRNA and confirmed knockdown efficiency by qPCR (Figure 5A). CCK-8 and Transwell assays showed that PSMG1 knockdown significantly inhibited tumor proliferation, invasion, and metastasis (Figure 5B-D). Additionally, the proliferation of BCa cells was reduced following PSMG1 knockdown (Figure 5E), and tumor volume was significantly decreased (Figure 5F), suggesting that elevated PSMG1 expression promotes BCa progression.

Given the close association between EMT and MIBC aggressiveness, we examined the effect of PSMG1 on EMT-associated proteins. Western blot results showed that knockdown of PSMG1 led to reduced expression of N-cadherin, Vimentin, and SNAIL, whereas E-cadherin levels were increased (Figure 5G). Among these EMT-related proteins, E-cadherin showed the clearest and most reproducible change after PSMG1 silencing. In light of this observation, together with the known function of the proteasome in protein turnover^51^, we investigated whether PSMG1 could influence E-cadherin stability. CHX chase analysis demonstrated that E-cadherin degradation was delayed in PSMG1-knockdown cells, resulting in a longer half-life than that observed in control cells (Figure 5H). These findings suggest that PSMG1 may promote EMT-related phenotypes by reducing E-cadherin stability.

### The H3K18la-PSMG1 axis was associated with BCa progression

**Figure 6:**
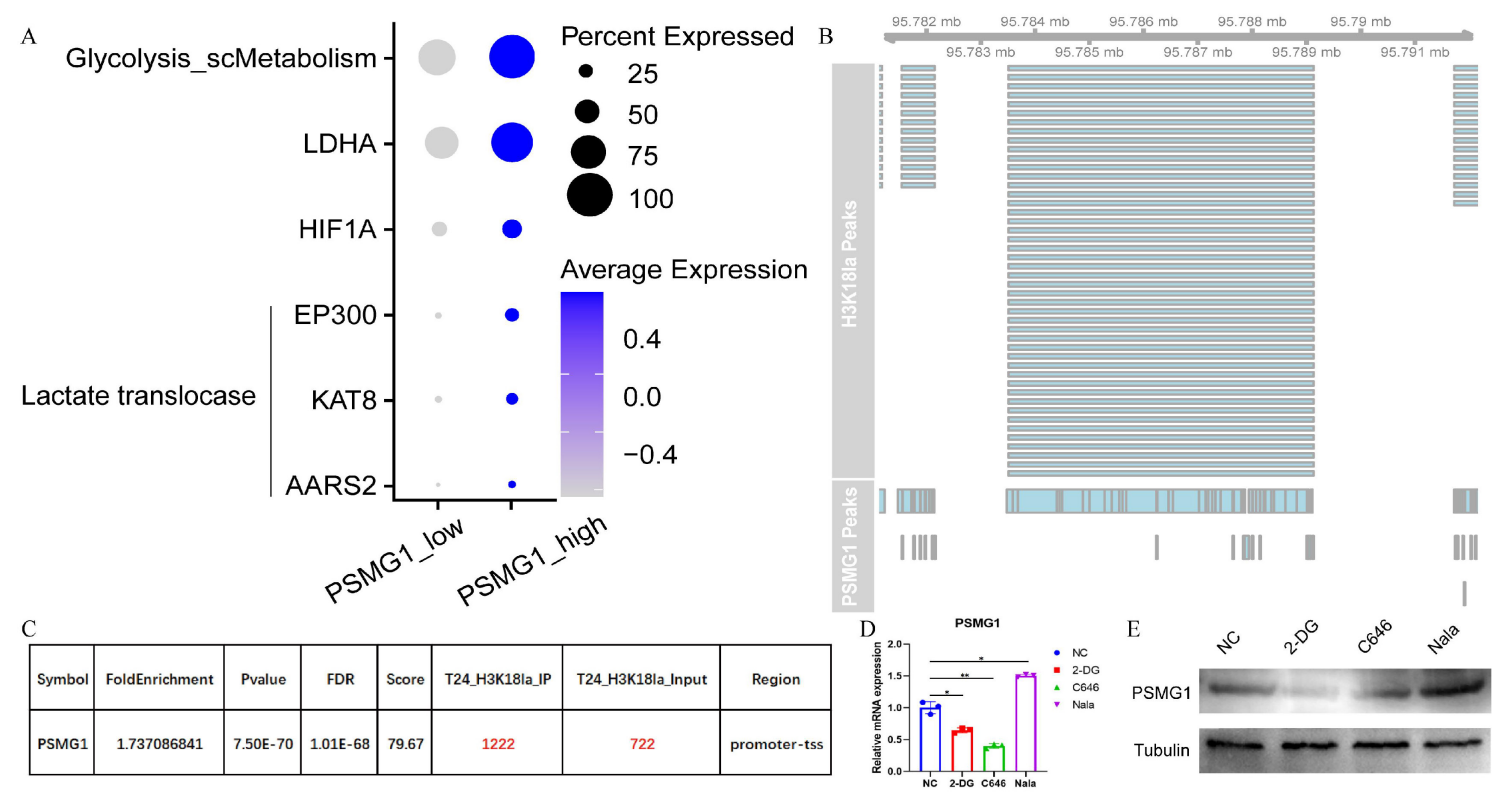
Evidence supporting an association between H3K18la and PSMG1 in BCa. **(A)** Bubble plot of glycolysis-related genes and lactate-associated factors between groups with high and low PSMG1 expression in tumor epithelial cells. **(B)** Visualization of chromatin open regions of H3K18la and PSMG1. **(C)** The enrichment of H3K18la sites was observed in the promoter region of PSMG1. **(D-E)** The changes in PSMG1 expression levels after the addition of histone lactylation inhibitors 2-DG and c646, and the histone lactylation promoter Nala, were validated using qPCR and Western blotting.

Histone lactylation is a recently recognized post-translational modification that plays a key role in gene expression regulation and tumor progression^52^-^54^. Consistent with enrichment analysis, single-cell metabolism profiling revealed enhanced glycolysis in tumor cells with high PSMG1 expression, along with elevated co-expression of lactate-associated factors such as EP300, KAT8, and AARS2 (Figure 6A). To further examine the potential link between lactylation and PSMG1 expression, we assessed publicly available histone H3K18la modification data. This analysis revealed overlapping chromatin open regions between H3K18la and PSMG1 (Figure 6B), suggesting a possible association between H3K18la enrichment and PSMG1 transcriptional regulation.

ChIP-seq analysis identified H3K18la-enriched regions in gene promoter areas, including the promoter region of PSMG1 (Figure 6C). These findings support a potential regulatory link between H3K18la and PSMG1 in BCa.

To further assess this association, we treated cells with histone lactylation inhibitors 2-DG and c646, which resulted in decreased PSMG1 mRNA expression, whereas treatment with the histone lactylation promoter Nala increased PSMG1 expression (Figure 6D). Western blotting showed similar trends at the protein level (Figure 6E). Collectively, these findings indicate that histone lactylation status correlates with PSMG1 expression, pointing to an H3K18la-PSMG1 regulatory axis in BCa. However, the direct causal mechanism requires further experimental validation.

### Curcumin was prioritized as a candidate compound associated with PSMG1 targeting in BCa

Natural products derived from traditional Chinese herbs have attracted increasing attention because of their potential anti-tumor effects^55^. These compounds have the potential to improve the effectiveness of chemotherapy, radiotherapy, and immunotherapy while mitigating treatment-related toxicities. They also lay a foundation for future therapeutic studies^56^-^59^.

**Figure 7:**
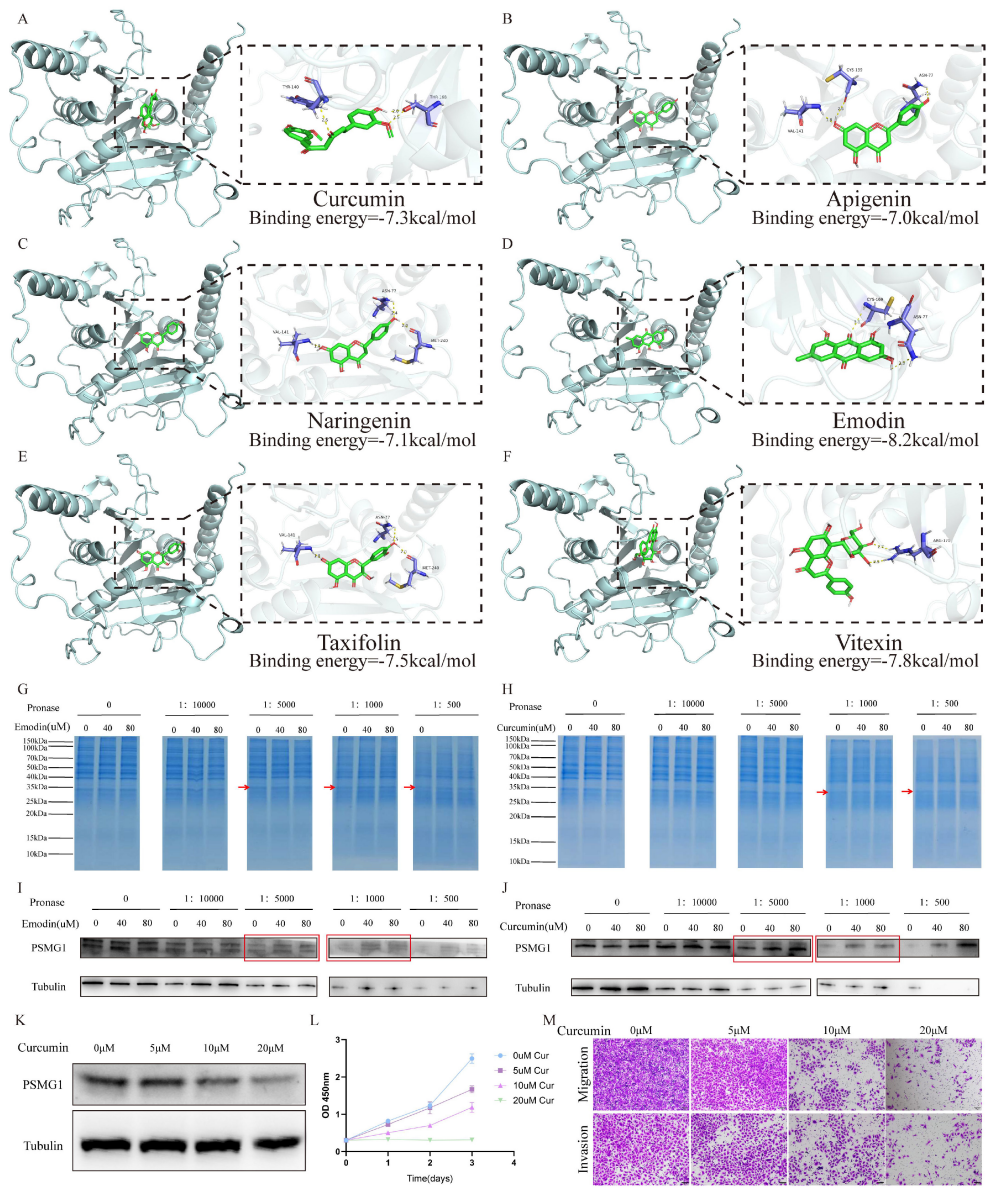
Screening and preliminary evaluation of candidate compounds associated with PSMG1. Molecular docking and DARTS assays were used as preliminary approaches to assess potential compound-PSMG1 interactions. For the docking results, interaction diagrams were generated using SwissDock and visualized with PyMOL and LigPlot+. Binding interfaces between drugs and PSMG1 were annotated to indicate hydrogen bonds, hydrophobic contacts, and relevant residues involved in the interaction. **(A-F)** Six drugs with their respective binding poses and interactions with PSMG1. From A to F, they were respectively: Curcumin, Apigenin, Naringenin, Emodin, Taxifolin, and Vitexin. **(G-H)** Coomassie blue staining of the DARTS assay. The regions corresponding to Emodin and Curcumin are shown at approximately 35 kDa.** (I-J)** Western blot analysis of the DARTS assay. Representative results are shown. **(K)** Protein expression of PSMG1 under different doses of Curcumin. **(L)** Effect of Curcumin on BCa cell proliferation assessed by CCK-8 assay. **(M)** Transwell assays showing the effects of Curcumin on BCa cell migration and invasion.

Using the Beyondcell algorithm, compounds associated with PSMG1-related drug sensitivity were identified. To estimate the binding potential of these candidate compounds, molecular docking analysis was performed using SwissDock. This analysis identified six candidate compounds with favorable binding poses and interaction energies with PSMG1 (Curcumin: -7.3 kCal/mol, Apigenin: -7.0 kCal/mol, Naringenin: -7.1 kCal/mol, Emodin: -8.2 kCal/mol, Taxifolin: -7.5 kCal/mol, and Vitexin: -7.8 kCal/mol), and the predicted interaction patterns were visualized (Figure 7A-F).

Among the shortlisted compounds, Emodin, which showed the lowest predicted binding energy, and Curcumin, a molecule with a favorable safety profile and documented anti-tumor activity, were selected for further investigation^58^, ^60^, ^61^. DARTS assays were subsequently performed to examine whether these compounds were associated with altered protease susceptibility of PSMG1. In the Emodin-treated group, degradation of the PSMG1-corresponding region was reduced relative to the control group (Figure 7G, I). A similar pattern was observed in Curcumin-treated cells (Figure 7H, J), supporting the possibility of an interaction between Curcumin and PSMG1. To further assess the biological relevance of Curcumin, we conducted proteomic sequencing (Supplementary Material 2). The sequencing data indicated decreased PSMG1 expression in Curcumin-treated cell lines, which was further supported by Western blot analysis (Figure 7K). Moreover, functional assays demonstrated that Curcumin inhibited BCa cell invasion and migration in a dose-dependent manner (Figure 7L-M). However, the present study did not directly establish that the observed decrease in PSMG1 expression was mechanistically caused by Curcumin-PSMG1 interaction. Overall, these findings support Curcumin as a prioritized candidate compound within the PSMG1-related therapeutic screening framework, while the mechanistic relationship between the putative Curcumin-PSMG1 interaction and reduced PSMG1 expression remains to be clarified.

## Discussion

PSMG1 (Proteasome Assembly Chaperone 1), also known as PAC1, is a chaperone protein involved in 20S proteasome assembly^51^. Our study identified PSMG1 as a key oncogene in BCa, showing progressive upregulation from normal tissue to NMIBC and MIBC, where it was associated with tumor aggressiveness and poor prognosis. Functional assays confirmed that PSMG1 promotes proliferation, invasion, and EMT, reinforcing its oncogenic role. Additionally, a prognostic model was developed from MIBC.RGs via multi-omics integration and machine learning, which effectively categorized BCa patients as high- or low-risk. Immune infiltration analysis further showed that the MIBC.RG-related prognostic risk pattern was associated with lower CD8⁺ T-cell infiltration and higher M2 macrophage infiltration, indicating a potential immunosuppressive state in advanced BCa. Nevertheless, our data do not directly demonstrate that PSMG1 regulates M2 macrophage polarization or CD8⁺ T-cell infiltration. Thus, these immune-related findings represent preliminary associations and do not constitute definitive proof that PSMG1 directly modulates the tumor immune microenvironment.

Our findings suggest that PSMG1 may contribute to BCa aggressiveness, at least partly, by affecting E-cadherin turnover and thereby facilitating EMT-related changes. In this study, E-cadherin was not identified through an unbiased substrate-screening approach. Instead, it was selected as a candidate downstream effector because analysis of EMT-associated markers showed that E-cadherin increased consistently after PSMG1 knockdown, whereas mesenchymal markers were reduced. CHX chase experiments further supported the notion that PSMG1 influences E-cadherin stability. In parallel, our data support a possible association between histone lactylation, particularly H3K18la, and PSMG1 expression. ChIP-seq analysis revealed H3K18la enrichment in the promoter region of PSMG1, while single-cell metabolic profiling showed that elevated PSMG1 expression was accompanied by increased glycolytic activity and higher expression of lactate-associated factors, including EP300, KAT8, and AARS2. Moreover, pharmacological modulation of histone lactylation altered PSMG1 expression at both the transcript and protein levels. Taken together, these findings suggest that glycolysis-related lactylation may participate in the transcriptional regulation of PSMG1 in BCa. However, because these observations are derived mainly from public epigenomic datasets and pharmacological perturbation, they should be regarded as supportive rather than definitive evidence of direct causality. Although H3K18la has previously been implicated in other malignancies^62^, our results extend these observations by suggesting a potential H3K18la-PSMG1 regulatory relationship in BCa.

Previous studies have indicated that Curcumin can modulate epigenetic and metabolic pathways, including histone acetyltransferase-related regulation and glycolytic activity^63^-^67^. In light of our results, these reports suggest that Curcumin may influence BCa progression through multiple interconnected mechanisms. Nevertheless, the relative importance of direct PSMG1 regulation compared with broader upstream effects remains unclear. Although molecular docking and DARTS assays in our study supported a possible interaction between Curcumin and PSMG1, we did not directly determine whether the reduction in PSMG1 expression observed after Curcumin treatment was caused by compound-protein interaction itself or by indirect regulatory events. We also did not directly assess E-cadherin expression following Curcumin treatment. Since Curcumin treatment was associated with reduced PSMG1 expression, and PSMG1 knockdown prolonged E-cadherin stability, it is conceivable that Curcumin may affect E-cadherin-related EMT signaling; however, this inference remains speculative and requires direct experimental confirmation. Future studies, including rescue experiments using PSMG1 overexpression after Curcumin exposure, as well as direct assessment of Curcumin's effects on epigenetic enzymes such as EP300 and KAT8 in BCa, will be necessary to clarify the underlying mechanism.

The clinical implications of these findings remain preliminary. Targeting histone lactylation, particularly H3K18la, may represent a potential strategy to suppress PSMG1-associated BCa progression. Curcumin, given its favorable safety profile and reported anticancer activity^58^, ^60^, ^61^, was prioritized in our screening framework as a candidate compound associated with reduced PSMG1 expression and attenuation of aggressive BCa phenotypes. In addition, combination strategies involving Curcumin and other therapeutic modalities, such as immune checkpoint inhibitors, epigenetic modulators, or cisplatin-based chemotherapy, may warrant further investigation in future preclinical studies^58^, ^68^, ^69^. However, the efficacy and mechanistic basis of such combinations were not evaluated in the present study.

We also acknowledge the limitations of this study. Although IHC validation from the Human Protein Atlas supports our findings, validation in larger independent cohorts (IHC or TMA) is lacking. Moreover, our functional evidence is derived from in vitro assays; xenograft and patient-derived organoid models will be essential for confirming Curcumin's therapeutic efficacy. Another key challenge is Curcumin's poor bioavailability due to low solubility and rapid metabolism^70^. Nanocarrier-based delivery systems^70^-^72^ (liposomes, nanoparticles, exosome encapsulation) and Curcumin derivatives (e.g., EF-24, morpholine-modified analogs)^73^ have shown improved pharmacokinetics and anticancer potency in preclinical models, and represent promising strategies for clinical translation.

In summary, our study identified PSMG1 as a key oncogene in BCa, supported a potential association between H3K18la and PSMG1 in BCa progression, and prioritized Curcumin as a candidate compound for further investigation in PSMG1-related therapeutic strategies.

## Conclusion

In this study, we found PSMG1 to be a key oncogenic driver in BCa progression and uncovered a potential link between the H3K18la-PSMG1 axis and BCa aggressiveness. Our findings suggest that histone lactylation, particularly H3K18la, may contribute to BCa aggressiveness in association with increased PSMG1 expression and subsequent activation of the EMT program. Furthermore, our data prioritized Curcumin as a candidate therapeutic compound associated with reduced PSMG1 expression and suppression of aggressive BCa phenotypes. These findings provide preliminary support for further investigation of Curcumin in the context of PSMG1-related therapeutic strategies.

## Supplementary Material

Supplementary figure.

Supplementary material 1 and 2.

Supplementary tables.

## Data Availability

The datasets presented in this study can be found in online repositories. The names of the repository/repositories and accession number(s) can be found in the article/supplementary material. All software applications used are included in this article. Relevant sequencing data and analysis data results can be downloaded from the attachment or obtained by contacting corresponding authors.

## Abbreviations

BCa: Bladder cancer
NMIBC: Non-muscle-invasive bladder cancer
MIBC: Muscle-invasive bladder cancer
MIBC.RGs: Muscle-invasive bladder cancer-related genes
PSMG1: Proteasome assembly chaperone 1
H3K18la: Histone H3 lysine 18 lactylation
EMT: Epithelial-mesenchymal transition
RNA-seq: RNA sequencing
scRNA-seq: Single-cell RNA sequencing
WGCNA: Weighted gene co-expression network analysis
TCGA: The Cancer Genome Atlas
GEO: Gene Expression Omnibus
CIBERSORT: Cell-type identification by estimating relative subsets of RNA transcripts
AUC: Area under the curve
ROC: Receiver operating characteristic
C-index: Concordance index
HR: Hazard ratio
OS: Overall survival
DFS: Disease-free survival
PFS: Progression-free survival
qPCR: Quantitative polymerase chain reaction
CHX: Cycloheximide
DARTS: Drug affinity responsive target stability
ChIP-seq: Chromatin immunoprecipitation sequencing
ATAC-seq: Assay for transposase-accessible chromatin using sequencing
KEGG: Kyoto Encyclopedia of Genes and Genomes
IHC: Immunohistochemistry
HPA: Human Protein Atlas
CCK-8: Cell Counting Kit-8
