# Supplementary material for: H3K18la-PSMG1 Axis in Bladder Cancer Progression: Curcumin as a Therapeutic Candidate
Source: Int J Biol Sci. 2026 May 29;22(11):5953–68. doi: 10.7150/ijbs.135180 (PMC13282777; doi:10.7150/ijbs.135180)
Supplement: Supplementary file 1 — Supplementary figure. [file ijbsv22p5953s1.pdf]

Supplementary Figure

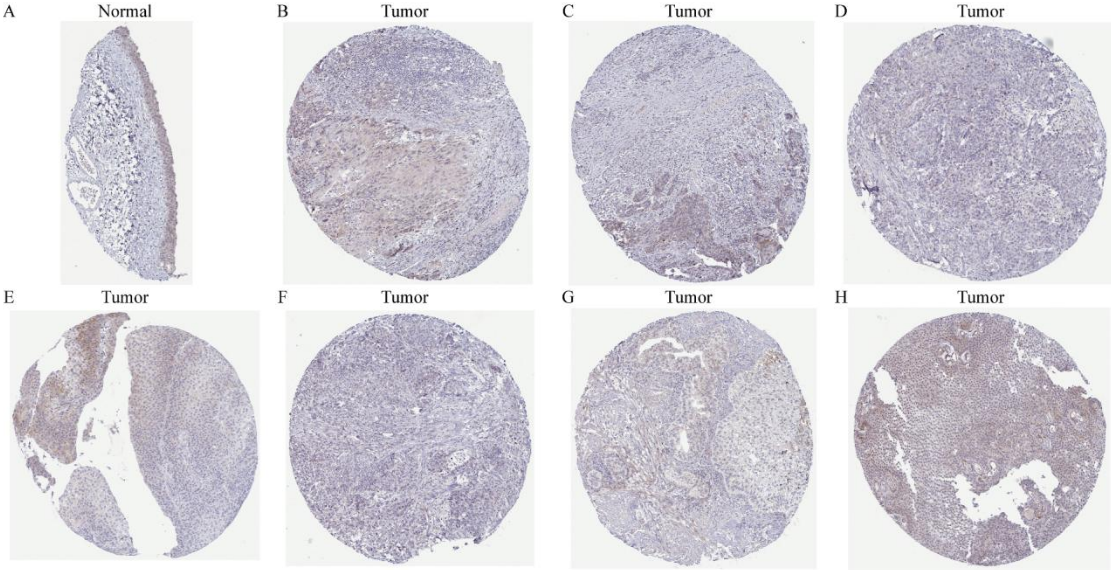

Figure S1

Immunohistochemistry image of PSMG1 expression levels in BCa from the HPA dataset. **(A)** Normal tissue, HPA057193, patient id: 1761. **(B)** Tumor tissue, HPA057193, patient id: 3079. **(C)** Tumor tissue, HPA057193, patient id: 3079. **(D)** Tumor tissue, HPA057193, patient id: 3111. **(E)** Tumor tissue, HPA057193, patient id: 4676. **(F)** Tumor tissue, HPA057193, patient id: 3111. **(G)** Tumor tissue, HPA057193, patient id: 4676. **(H)** Tumor tissue, HPA057193, patient id: 4678.
