# Supplementary material for: H3K18la-PSMG1 Axis in Bladder Cancer Progression: Curcumin as a Therapeutic Candidate
Source: Int J Biol Sci. 2026 May 29;22(11):5953–68. doi: 10.7150/ijbs.135180 (PMC13282777; doi:10.7150/ijbs.135180)
Supplement: Supplementary file 2 — Supplementary material 1 and 2. [file ijbsv22p5953s2.zip › Supplementary Material 1-Machine Learning.docx]

The machine learning-based integration approach employed in this study was adapted from previously published work by Chen et al., 2022 (**PMID: 35145098**), which developed an immune-derived lncRNA signature for colorectal cancer prognosis. In our study, we implemented a similar strategy to identify key genes associated with muscle-invasive bladder cancer (MIBC) and construct a robust prognostic model.

**1. Data Preprocessing and Feature Selection**

Prior to model development, raw gene expression data from multiple bulk RNA-seq datasets underwent standard preprocessing steps:

Normalization: Log2 transformation and z-score normalization were applied to standardize gene expression values.

Batch Effect Correction: The ComBat function from the sva package in R was used to eliminate potential batch effects among datasets.

Univariate Cox Regression Analysis: An initial screening was performed using univariate Cox regression (p < 0.05) to select genes significantly associated with overall survival.

**2. Machine Learning Algorithm Integration**

To improve the robustness of the prognostic model, we integrated ten machine learning algorithms for feature selection and survival prediction:

Random Survival Forest (RSF): RSF is an ensemble learning method based on decision trees, specifically designed for survival analysis with censored data. It evaluates the importance of each variable by generating multiple randomized decision trees, improving model robustness.

CoxBoost: CoxBoost is an adaptation of the Cox proportional hazards model using boosting techniques. It is particularly effective for high-dimensional survival data, selecting the most relevant predictors while minimizing overfitting.

Elastic Net (Enet): Elastic Net combines LASSO (L1 regularization) and Ridge (L2 regularization), allowing for automatic feature selection while retaining the advantages of Ridge regression in handling correlated predictors.

LASSO Regression: LASSO (Least Absolute Shrinkage and Selection Operator) applies L1 regularization to shrink some regression coefficients to zero, effectively eliminating irrelevant variables and enhancing model interpretability.

Stepwise Cox Regression: Stepwise Cox regression is an iterative method that adds or removes variables based on their statistical significance, constructing an optimized Cox proportional hazards model to identify the most relevant prognostic factors.

Ridge Regression: Ridge regression applies L2 regularization, which helps prevent overfitting in datasets with multicollinearity, making it suitable for high-dimensional gene expression data.

Gradient Boosting Machine (GBM): GBM is a boosting-based ensemble learning technique that sequentially builds weak models to minimize prediction errors. It enhances survival prediction performance by capturing complex relationships between variables.

Partial Least Squares Regression for Cox (plsRcox): PlsRcox integrates partial least squares (PLS) regression with Cox survival analysis, making it ideal for datasets where the number of predictors exceeds the number of samples.

Survival-Support Vector Machine (survival-SVM): Survival-SVM extends support vector machines (SVMs) for survival analysis, effectively capturing nonlinear relationships in high-dimensional survival data.

Supervised Principal Components (SuperPC): SuperPC applies principal component analysis (PCA) with supervised learning to reduce dimensionality while preserving key survival-related features, improving computational efficiency and model stability.

**3. Model Optimization and Validation**

Feature Selection: Each algorithm generated an independent feature ranking. To ensure robustness, we retained genes consistently selected across multiple algorithms.

Risk Score Calculation: The final prognostic model was built using the Ridge regression algorithm, which achieved the highest concordance index (C-index = 0.673). A risk score was computed for each patient using the weighted expression values of the selected genes.

Cross-Validation: The model’s performance was assessed using 10-fold cross-validation to prevent overfitting.

Survival Analysis: Kaplan-Meier survival curves, log-rank tests, and receiver operating characteristic (ROC) curve analysis were employed to evaluate the predictive accuracy of the model.

External Validation: The prognostic model was validated across multiple independent datasets from GEO and TCGA, demonstrating its robustness and generalizability.

**4. Comparison with Previous Studies**

Compared to conventional single-algorithm models, this multi-algorithm integration significantly improved:

Feature Selection Accuracy: The combination of different models reduced bias and improved gene selection stability.

Predictive Performance: The ensemble approach enhanced survival prediction accuracy across different patient cohorts.

Interpretability: By leveraging multiple algorithms, the model provided deeper biological insights into MIBC-associated gene signatures.

**5. Conclusion**

This machine learning-based integration framework offers a highly effective strategy for biomarker discovery and prognostic modeling in cancer research. By integrating multiple algorithms, our study successfully identified PSMG1 as a key regulator of BCa progression and constructed a predictive model with strong clinical relevance.
